# Impact of individualized management of breakthrough cancer pain on quality of life in advanced cancer patients: CAVIDIOPAL study

**DOI:** 10.1007/s00520-021-06006-1

**Published:** 2021-02-03

**Authors:** Albert Tuca Rodríguez, Miguel Núñez Viejo, Pablo Maradey, Jaume Canal-Sotelo, Plácido Guardia Mancilla, Sonia Gutiérrez Rivero, Inmaculada Raja Casillas, María Herrera Abián, Cristina López Bermudo

**Affiliations:** 1 Hospital Clínic i Provincial, Barcelona, Spain; 2 University of Barcelona, Barcelona, Spain; 3 Complejo Hospitalario Universitario de Orense, Ourense, Spain; 4 Hospital de Sant Joan de Déu, Palma de Mallorca, Spain; 5 Hospital Universitari Arnau de Vilanova, Lleida, Spain; 6 Hospital San Cecilio, Granada, Spain; 7 Hospital Universitario Virgen del Rocío, Sevilla, Spain; 8 Hospital Virgen de la Salud, Toledo, Spain; 9 Hospital Universitario Fundación Jiménez Díaz, Madrid, Spain; 10 Medical Department, Angelini Pharma España S.L.U., Barcelona, Spain

**Keywords:** Breakthrough cancer pain, Quality of life, Transmucosal fentanyl, Individualized therapy, Palliative care

## Abstract

**Purpose:**

The main aim of the study was to assess the impact of individualized management of breakthrough cancer pain (BTcP) on quality of life (QoL) of patients with advanced cancer in clinical practice.

**Methods:**

A prospective, observational, multicenter study was conducted in patients with advanced cancer that were assisted by palliative care units. QoL was assessed with the EORTC QLQ-C30 questionnaire at baseline (V0) and after 28 days (V28) of individualized BTcP therapy. Data on background pain, BTcP, comorbidities, and frailty were also recorded.

**Results:**

Ninety-three patients completed the study. Intensity, duration, and number of BTcP episodes were reduced (*p* < 0.001) at V28 with individualized therapy. Transmucosal fentanyl was used in 93.8% of patients, mainly by sublingual route. Fentanyl titration was initiated at low doses (78.3% of patients received doses of 67 μg, 100 μg, or 133 μg) according to physician evaluation. At V28, mean perception of global health status had increased from 31.1 to 53.1 (*p* < 0.001). All scales of EORTC QLQ-C30 significantly improved (*p* < 0.001) except physical functioning, diarrhea, and financial difficulties. Pain scale improved from 73.6 ± 22.6 to 35.7 ± 22.3 (*p* < 0.001). Moreover, 85.9% of patients reported pain improvement. Probability of no ≥ 25% improvement in QoL was significantly higher in patients ≥ 65 years old (OR 1.39; 95% CI 1.001–1.079) and patients hospitalized at baseline (OR 4.126; 95% CI 1.227–13.873).

**Conclusion:**

Individualized BTcP therapy improved QoL of patients with advanced cancer. Transmucosal fentanyl at low doses was the most used drug.

**Trial registration:**

This study was registered at ClinicalTrials.gov database (NCT02840500) on July 19, 2016.

**Supplementary Information:**

The online version contains supplementary material available at 10.1007/s00520-021-06006-1.

## Introduction

Breakthrough cancer pain (BTcP) is frequent in cancer patients and it significantly impairs their quality of life (QoL) [1–3]. It is usually defined as a transient pain exacerbation, which is spontaneous or triggered, in spite of a stable and adequately controlled background pain. In addition, it should be differentiated from the “end of dose” pain [4]. Its prevalence ranges from 19 to 95% according to different studies [5]. In a systematic review with 19 studies, mean prevalence of BTcP was 59.2%. Prevalence was higher in palliative care units (hospices) than in outpatient clinics (80.5% and 39.9%, respectively) [2]. Moreover, in observational studies, BTcP was predictable in 30–44% of patients, achieved its peak intensity in less than 10 min in more than 60% of episodes, had a mean duration of 30 min, and usually interfered with patient QoL [3, 6, 7].

BTcP requires appropriate diagnosis and individualized management [8]. An algorithm, developed by Portenoy et al. [1] and modified by Davies et al. [4], is useful for BTcP diagnosis. According to this algorithm, the patient should have background cancer-related pain, defined as pain for at least 12 h during the previous week, or pain that is present when he/she is not taking analgesics. Moreover, background pain should be adequately controlled (it should be none or mild but not moderate or severe during more than 12 h during the previous week). Finally, the patient should have transient exacerbations of pain with at least moderate intensity [4]. Once diagnosed, BTcP should be treated with effective drugs, with dose and administration route appropriated to the type of pain. However, it is important to adapt therapy to specific needs of each patient [9].

Individualized BTcP management consists of an assessment process, from pain diagnosis to follow-up. First, physicians should assess patient characteristics and pain features (triggers, intensity, duration, and frequency). Adherence to background pain therapy should also be assessed. After BTcP diagnosis, strong opioid medication should be prescribed, considering the patient needs and preferences. Currently, specific guidelines support the use of rapid-onset opioids (ROOs) as rescue medication in unpredictable and rapid-onset BTcP, and the use of standard normal-release oral opioids in those cases with slow-onset pain episode or as pre-emptive administration before a predictable pain episode triggered by known events [8, 10]. ROOs are based on transmucosal fentanyl citrate, because its pharmacokinetics profile fits BTcP characteristics (high intensity, rapid-onset, and short duration) better than oral morphine [8, 11, 12]. ROO dose should be carefully titrated for each patient, until finding the dose that is efficacious and well tolerated with minimum side effects. If possible, the route of administration should be chosen after informing the patient and caregivers of the advantages and disadvantages of each route. Thereafter, according to the guidelines, BTcP management should be reassessed regularly. In addition, adherence and potential adverse events should also be evaluated periodically [8, 9].

Patients with advanced cancer assisted in palliative care units had multiple symptoms, chronic comorbidities, impaired functional and health status, and very limited life expectancy. Because of their frailty profile, these patients are usually excluded from clinical trials of BTcP management. Moreover, there are few data on QoL in these patients. Therefore, the main aim of the CAVIDIOPAL study was to assess QoL of patients with advanced cancer and BTcP treated with individualized pain therapy in palliative care units. Secondary aims included to examine BTcP features, global impression of improvement after individualized BTcP management, and frailty and its potential effect on QoL.

## Methods

### Study design

A prospective, observational, multicenter study was conducted in patients with advanced cancer who were assisted by Spanish Palliative Care teams.

### Study population

Both hospitalized patients and outpatients were recruited from palliative care units at acute care hospitals in Spain. Inclusion criteria were age ≥ 18 years, diagnosis of advanced cancer, clinically estimated survival ≥3 months, attendance by palliative care units, background cancer pain controlled with opioids, diagnosis of BTcP using the Davies algorithm [4], and signed informed consent.

Patients were excluded if they had severe cognitive impairment or other condition or disease that hampered the study understanding or data collection according to the investigator judgment. Patients were also excluded if they fulfilled criteria of addiction to opioids, alcohol, or other recreational drugs.

### Measures

Pain intensity was measured with a visual analog scale (VAS) from 0 (no pain) to 11 (unbearable pain) points. Moderate pain intensity was defined as a value ≥ 4 in VAS.

At baseline (V0, first visit), after obtaining signed informed consent, BTcP treatment was individualized. This included a careful assessment of BTcP episodes; information on pain features; advisement on non-pharmacological therapy; onset or change of analgesia for BTcP episode according to the physician judgment; education on prescribed drug use; follow-up of BTcP therapy at each visit; alert measures; and adverse event prevention. An integral approach was also proposed, which included management of the rest of symptoms, and psychosocial and spiritual support, according to specific needs of patient.

Primary outcome was QoL, measured with the European Organization for Research and Treatment of Cancer Quality of Life Questionnaire Core 30 items (EORTC QLQ-C30) version 3.0 [13] at baseline (V0) and after 28 days (V28) of individualized BTcP therapy. This questionnaire has been validated for its use in Spain [14]. It consists of 30 items, composed of both multi-item scales and single-item measures, distributed in one global health status/QoL scale, five functional scales (physical, role, emotional, cognitive, and social), three symptom scales (fatigue, pain, and nausea and vomiting), and six single items (dyspnea, insomnia, anorexia, constipation, diarrhea, and financial difficulties). High score for global health status means a high QoL, and high scores for functional scale indicate a high level of functioning. However, high scores for symptom scale or single items represent a high symptom burden and low QoL [13].

The other study variables were measured at baseline visit (V0) and at the visits 8 (V8), 15 (V15), 28 (V28), and/or 90 (V90) days after inclusion in the study. An ad hoc questionnaire to assess the study variables was developed. In addition of the EORTC QLQ-C30, the questionnaire was composed of socio-demographic data (V0); type of cancer and its stage (V0); global health status measured with the Eastern Cooperative Group performance status scale (ECOG-PS) [15] (V0, V28, and V90); comorbidities according to the Charlson index [16] (V0); nutritional status and cachexia according to the Evans criteria [17] (V0); occurrence of dry mouth (V0); cognitive status measured with the Pfeiffer test [18] (V0); and symptom burden according to the Edmonton Symptom Assessment System (ESAS) [19] (V0). The questionnaire also assessed type of background pain and its intensity measured with a 11-point VAS (V0, V8, V15, V28, and V90); type, number, duration, and intensity (VAS) of BTcP episodes (V0, V8, V15, V28, and V90); opioid treatment for background pain (V0, V8, V15, V28, and V90); adjuvant analgesic therapy (V0 and V28); non-pharmacological therapy (V0, V8, V15, V28, and V90); and opioid therapy for BTcP (V0, V8, V15, V28, and V90). Other variables evaluated were falls during the previous month (V0 and V28); hospitalization or emergency room visits during the previous month (V28); and treatment withdrawal by opioid toxicity during the previous month (V28). Finally, the questionnaire included the Patient Global Impression of Improvement (PGI-I) scale (V28) and the Clinical Global Impression of Improvement (CGI-I) scale (V28). PGI-I and CGI-I are Likert scales of 7 points, from 0 (very much improved) to 7 (very much worse) [20].

### Statistical analysis

A descriptive analysis of all the registered variables was performed. Global health status and EORTC QLQ-C30 scores at V0 and V28 were compared. In addition, the BTcP-related variables and BTcP treatment at each visit were analyzed. Moreover, EORTC QLQ-C30 results were stratified by frailty variables (ECOG score, age, hospitalization at baseline, cognitive status, falls, malnutrition, and comorbidities).

Categorical variables were expressed with number and percentage, and continuous variables with mean (± standard deviation) and median (95% confidence interval). The Student *t* test or the Wilcoxon signed-rank test was used for analyzing differences between categorical variables, while the chi-squared test or the Fisher test was employed for differences between continuous variables. In addition, to identify variables of frailty related to changes in QoL, an analysis of covariance (ANCOVA) was performed; an increase ≥ 25% in the EORTC QLQ-C30 score between V0 and V28 was considered as an improvement in QoL. Then, the variables with differences with *p* value < 0.1 were included in a logistic regression analysis.

All statistical analyses were conducted using IBM SPSS Statistics V22.0. *p* values <0.05 were considered statistically significant. However, all the statistical tests performed are explorative and descriptive since the sample size was not calculated. To our knowledge, there were no previous studies that identified the magnitude of variations in QoL parameters related to simple factors of frailty. Therefore, we were not able to estimate the sample size. A population of at least 100 patients was the sample objective, considering the recruitment ability and that the study was carried out in the clinical practice framework.

## Results

### Characteristics of study population

From June 2016 to December 2017, 100 patients were recruited from 8 out of the 10 planned palliative care units. One patient did not have advanced cancer and was excluded. Written informed consent was obtained from all individual participants in the study (*n* = 99). Five patients died before V28, one patient withdrawn consent, and 93 patients completed the study until V28.

Most patients were men and mean age was 69 years. Almost 75% of patients were older than 60 years and more than 25% of them were over 80 years old. More than 70% of patients were outpatients. The most common locations of the primary tumor were the pancreas, lung, breast, and colon, while metastases (present in more than 80% of patients) were located more frequently in the bone, liver, lung, and lymph nodes. At baseline, most patients (77.7%) had an ECOG-PS score ≤ 2. Comorbidities were common, especially diabetes mellitus, chronic obstructive pulmonary disease, and chronic heart failure. Few patients presented mild cognitive impairment (7%) at baseline. Moreover, 24% met criteria of cachexia. Almost half of patients complained of dry mouth. Finally, no patient showed addictive behavior (Table 1).

**Table 1:** Baseline characteristics of patients (*N* = 99)

Gender, *N* (%)	
Female	38 (38.4)
Male	61 (61.6)
Age, mean (SD)	69.0 (13.0)
< 60 years	26 (26.3)
60–70 years	29 (29.3)
70–80 years	19 (19.2)
> 80 years	25 (25.3)
Hospital status, *N* (%)	
Outpatients	71 (71.7)
Inpatients	28 (28.3)
Primary cancer, *N* (%)	
Pancreas	16 (16.2)
Lung	13 (13.1)
Breast	9 (9.1)
Colon	8 (8.1)
Gastric	7 (7.1)
Prostate	6 (6.1)
Bladder	6 (6.1)
Other	34 (34.2)
Stage	
II	6 (6.1)
III	13 (13.1)
IV	80 (80.8)
Location of metastases*, *N* (%)	
Bone	35 (35.4)
Liver	24 (24.2)
Lung	21 (21.2)
Ganglia	16 (16.2)
Other	23 (23.1)
Functional status (ECOG), *N* (%)	
0	13 (13.1)
1	34 (34.3)
2	30 (30.3)
3	21 (21.2)
4	1 (1.0)
Comorbidities**	
Charlson index, mean (SD)	6.5 (2.0)
Diabetes mellitus, *N* (%)	20 (20.2)
Chronic obstructive pulmonary disease, *N* (%)	17(17.2)
Chronic heart failure, *N* (%)	12 (12.1)
Chronic liver disease, *N* (%)	8 (8.1)
Other chronic conditions, *N* (%)	39 (39.4)
Cachexia, *N* (%)	24 (24.2)
Dry mouth, *N* (%)	46 (46.5)
Addictive behavior, *N* (%)	0 (0)
Cognitive status	
No cognitive impairment	92 (92.9)
Mild cognitive impairment (Pfeiffer test ≤4)	7 (7.1)
Falls over the previous month, mean (SD)	0.2 (0.5)

*SD*, standard deviation; *ECOG*, Eastern Cooperative Oncology Group scale

*Patients could have metastases in more than one location

**Patients could have more than one comorbidity

Background pain had a mean intensity of 4 at baseline. Transdermal fentanyl was the main treatment, used in more than 45% of patients and with a mean dose of 81.1 μg/h. Oral morphine was employed in more than 15% of patients. Considering all opioids administered, mean oral morphine equivalent dose was 110.5 ± 46.7 mg/day. Other therapies for background pain were adjuvant drugs, mainly anticonvulsants and antidepressants (Table 2).

**Table 2:** Background pain and breakthrough cancer pain at baseline (*N* = 99)

Background pain	
Intensity (VAS score), mean (SD)	4.0 (1.9)
Opioid therapy, *N* (%)	
Transdermal fentanyl	45 (45.5)
Oral morphine	15 (15.2)
Oxycodone-naloxone	13 (13.1)
Oral oxycodone	11 (11.1)
Tramadol	7 (7.1)
Tapentadol	7 (7.1)
Methadone	1 (1.0)
Adjuvant drugs*, *N* (%)	
Anticonvulsants	32 (32.3)
Antidepressants	20 (20.2)
Benzodiazepines	14 (14.1)
NSAIDs	4 (4.0)
BTcP episodes	
Type, *N* (%)	
Visceral nociceptive	20 (20.2)
Somatic nociceptive	18 (18.2)
Neuropathic	3 (3.0)
Mixed	58 (58.8)
Trigger factor, *N* (%)	
Spontaneous	50 (50.5)
Incident	49 (49.5)
Volitional	21 (21.2)
Non volitional	20 (20.2)
Procedural	8 (8.1)
Episodes of BTcP during the previous 24 h, mean (SD)	3.6 (1.6)
Intensity (VAS score), mean (SD)	8.0 (1.0)
Duration in minutes, mean (SD)	27.5 (15.9)
Opioid therapy	
Transmucosal fentanyl	93 (93.8)
Oral morphine	5 (5.1)
Oral oxycodone	1 (1.0)
Adjuvant non-drug therapy, *N* (%)	17 (17.2)

*BTcP*, breakthrough cancer pain; *NSAID*, nonsteroidal anti-inflammatory drug; *SD*, standard deviation; *VAS*, visual analog scale

*Patients could receive more than one adjuvant therapy

### BTcP and background pain evolution

At baseline, BTcP was spontaneous in nearly 50% of patients and incident in the other 50% of patients. It had a mean intensity of 8 and it was located mainly in the abdomen (42% of patients) and chest (18% of patients). Patients suffered a mean of 3.6 episodes a day, with a mean duration of 27.5 min per episode. Therefore, the mean time with uncontrolled pain (number of episodes multiplied by its duration) per patient was 99 ± 20.2 min/day. Transmucosal fentanyl, especially by sublingual route, was the most used opioid (Table 2).

BTcP therapy was individualized for all patients. This descriptive study did not specify the type or dose of the rescue pain reliever for BTcP, so this should be according to clinical practice. The only condition was the use of a ROO or a short-acting opioid for the treatment of BTcP crisis, in doses proportional or not to the dose of the opioid for background pain, according to the criteria of the treating physician, administered at intervals not less than 4 h and with close monitoring of the analgesic effect and its tolerance to proceed to a progressive dose titration. Dose was adjusted or treatment was initiated with transmucosal fentanyl in 93 patients (93.9%), oral standard release morphine in 5 patients (5.1%), and standard release oxycodone in 1 patient (1.0%). Mean initial dose of transmucosal fentanyl prescribed at V0 was 116.7–± 86.2 μg, with a minimum dose of 67 μg in 39 patients and a maximum dose of 400 μg in 6 patients, according to the physician evaluation. Moreover, 17 patients also received non-pharmacological therapy, including physical therapy (5.1%), radiation therapy (4.0%), psychotherapy (4.0%), and nerve block (1.0%). Despite 35% of patients presented bone metastasis, only 4% were treated with radiotherapy, because in most of cases, this treatment was administered before inclusion in this study.

Intensity of pain (VAS scores) was reduced significantly at V8, V15, V28, and V90 compared with V0 (*p* < 0.001 for each visit), both for background pain and BTcP. Mean intensity of background pain was reduced from 4 at V0 to 2 at V28 and 1.8 at V90 (*p* < 0.001). Mean intensity of BTcP was reduced from 8 ± 1.0 at V0 to 4.6 ± 2.4 at V28 and 4 ± 2.4 at V90 (*p* < 0.001).

Opioid therapy for background pain was changed in 45.9% of patients at V8, with successive reductions in the number of patients with opioid change. Most of these changes were dose adjustments (V8, 34.3%; V15, 20.2%; V28, 1.1%). Opioid therapy for BTcP was also changed in 38.8% of patients at V8 and 12.6% at V15, but then 93 patients (97.9%) did not have therapies changes from V28 to V90 (*p* < 0.001). Dose adjustments were the main cause of these changes in analgesia (74.5%), which was reduced during the study.

Number and duration of BTcP episodes diminished during the study, as well as time until acceptable pain relief and time with uncontrolled pain. See Table S1 (Online Resource 1) for detailed data.

Between V0 and V28, 12 patients (12.1%) required hospitalization, including one case of non-predicted opioid side effect (acute urinary retention).

### Primary outcome: quality of life

At baseline, mean EORTC QLQ-C30 score was 31 ± 22.4. Scores of EORTC QLQ-C30 scales ranged from a mean maximum value of 73.6 ± 22.6 for pain to a mean minimum value of 5.1 ± 14.6 for diarrhea. After 28 days with individualized BTcP therapy, there was a statistically significant improvement in global health status, role, emotional, cognitive and social functioning, fatigue, pain, nausea and vomiting, dyspnea, insomnia, appetite loss, anorexia (*p* < 0.001 for each item), and constipation (*p* = 0.001). In particular, the pain rating changed from a mean of 73.6 ± 11.6 at V0 to a mean of 37.7 ± 22.3 at V28 (*p* < 0.001), indicating a significant reduction. No statistically significant differences were found for physical functioning, diarrhea, and financial difficulties (Table 3).

**Table 3:** EORTC QLQ-C30 score at baseline and after 28 days of individualized therapy of BTcP in patients with advanced cancer

EORTC QLQ-C30	D0 mean (SD)	D28 mean (SD)	*p*
Global health status	31.1 (22.4)	53.1 (22.8)	< 0.001
Functional scales			
Physical functioning	49.8 (32.9)	55.5 (29.1)	0.114
Role functioning	34.8 (30.4)	47.0 (28.8)	< 0.001
Emotional functioning	49.7 (26.8)	68.5 (23.5)	< 0.001
Cognitive functioning	65.8 (25.4)	81.2 (23.6)	< 0.001
Social functioning	37.9 (31,8)	54.3 (29.6)	< 0.001
Symptom scales			
Fatigue	56.6 (26.5)	44.2 (22.1)	< 0.001
Pain	73.6 (22.6)	35.7 (22.3)	< 0.001
Nausea-vomiting	13.5 (21.6)	4.7 (12.4)	< 0.001
Single items			
Dyspnea	21.5 (28.3)	11.5 (19.3)	< 0.001
Insomnia	47.1 (31.9)	16.8 (22.9)	< 0.001
Appetite loss	44.1 (29.7)	18.6 (21.1)	< 0.001
Constipation	34.7 (30.8)	24.7 (24.0)	< 0.001
Diarrhea	5.1 (14.6)	2.9 (11.7)	0.278
Financial difficulties	31.3 (30.8)	26.5 (29.3)	0.055

*EORTC QLQ-C30*, European Organization for Research and Treatment of Cancer Quality of Life Questionnaire Core 30 items; *BTcP*, breakthrough cancer pain; *SD*, standard deviation

### Global impression of improvement

**Fig. 1:**
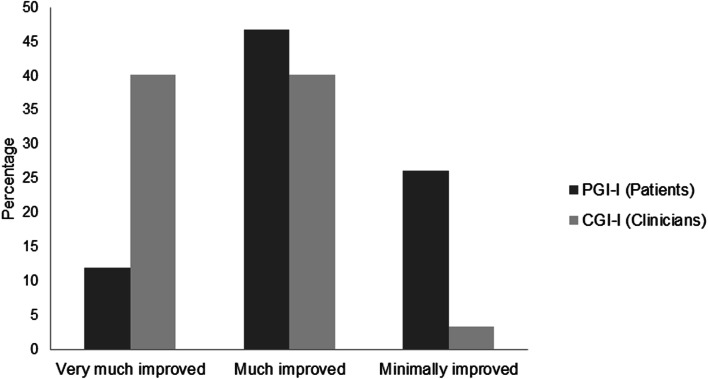
PGI-I and CGI-I reporting improvement after 28 days of individualized BTcP therapy in patients with advanced cancer and BTcP assisted in palliative cancer units (*N* = 92). PGI-I, patient global impression-improvement; CGI-I, clinical global impression-improvement; BTcP, breakthrough cancer pain

After 28 days of individualized BTcP therapy, 84.8% of patients and 83.7% of physicians reported some grade of global impression of improvement. “Very much improved” or “much improved” was reported by 58.6% of patients and 80.4% of physicians (Fig. 1).

### Factors influencing quality of life after individualized approach of BTcP

A univariate analysis was performed to compare patients with ≥ 25% improvement in the EORTC QLQ-C30 score stratified by symptom burden, variables related with frailty (age, falls in the previous month, ECOG-PS score, hospitalization at inclusion, cachexia, or dry mouth), and pain characteristics (background pain and BTcP intensity at baseline, BTcP characteristics, or BTcP therapy and adjuvant analgesia).

Sixty patients (60.6%) presented a high symptom burden, defined as ≥ 5 symptoms according the ESAS instrument, with at least moderate intensity. After 28 days, no statistically significant difference in the EORTC QLQ-C30 score (*p* < 0.211) was found between patients with and without high symptom burden. However, when adjusting by the symptom burden at baseline, difference was statistically significant (*p* = 0.008).

Patients with ≥ 25% improvement in the EORTC QLQ-C30 questionnaire were compared according to a series of variables. For the variable “age,” patients were categorized as ≤ 65 years old (40.9% of patients) and > 65 years old (59.1% of patients). Only age, number of BTcP episodes during the previous 24 h, and hospitalization at study inclusion were identified as significant (*p* < 0.1) for use in the logistic regression model. However, the variable “number of BTcP episodes” was excluded because it was not significant in the model.

Results showed that probability of no QoL improvement was significantly higher (*p* < 0.05) with age > 65 years and hospitalization. The risk of no improvement was 1.04-fold increased for each year of age above 65 and 4.06-fold increased with hospitalization at baseline (Table 4).

**Table 4:** Logistic regression analysis: risk of no improvement in patients with advanced cancer and BTcP with individualized pain therapy

Variable	B	SE	Wald	df	Sig.	OR	95% CI for OR	
							Lower	Upper
Age	0.038	0.019	3.949	1	0.047	1.039	1.001	1.079
Hospitalization	1.417	0.619	5.246	1	0.022	4.126	1.227	13.873
Constant	− 4.615	1.566	8.687	1	0.003	0.010	-	-

## Discussion

The impact on QoL of individualized BTcP therapy in patients with advanced cancer assisted in palliative care units was examined. Socio-demographic data and clinical characteristics of patients were similar to those collected in previous studies [3]. Pancreatic cancer was the most common, because some investigators were involved in early palliative interventions for patients with this tumor in advanced stages. Moreover, pancreatic cancer is one of the most frequent causes of oncological pain [21]. Davies algorithm [4] was chosen for BTcP diagnosis in the protocol study, because it is the most widely accepted in guidelines for oncological pain [8, 11, 22]. BTcP intensity, duration, and number of episodes, as well as BTcP impact on QoL, were similar to those found in previous studies using the same diagnostic criteria [3, 7, 23, 24].

After individualized BTcP approach, intensity of background pain and BTcP, number of episodes, and time to response were reduced, and hence the daily time with poor controlled pain. Considering that in this study, most patients had a rapid-onset and short duration pain episode, and at least half of the patients had spontaneous BTCP, and as recommended in guidelines [10–12, 25, 26], the transmucosal fentanyl was the most common ROO administered. Moreover, expert consensuses recommend initiating BTcP therapy at low dose in frail patients, and then carefully titrate dose based on efficacy and tolerability [27–29]. In many patients of this study, because of their advanced age and frailty, and according to the physician evaluation, fentanyl was titrated starting from the lowest available dose (67 μg). ROO dose was increased in more than 30% of patients at V8, but only in 12% of patients at V28. In addition, opioid therapy was well tolerated.

The EORTC QLQ-C30 questionnaire was used because it is specific for cancer. Furthermore, it is the QoL questionnaire most used in clinical trials in Europe and is validated for the Spanish population [14]. It allows assessing physical, emotional, and social aspects of QoL of patients with cancer, as well as their general functionality [13]. Patients with BTcP have reported poor QoL with low physical, psychological, and social well-being [30]. In the CAVIDIOPAL study, and as expected, EORTC QLQ-C30 scores were lower than those in the Spanish general population [31]. However, after individualized BTcP therapy and the resulting pain relief, patients experienced a statistically significant increase in their QoL. Improvement was statistically significant in global health status as well as in almost all subscales and items of the EORTC QLQ-C30 questionnaire. Improvement in pain was considerable, with almost 30 points of difference in the mean score between baseline and after 28 days of individualized BTcP therapy. Despite the particular improvement on the subscale of pain of the EORTC QLQ-C30, we cannot rule out that overall improvement of QoL was also due to a comprehensive approach of patient’s care, which includes control of the other symptoms, and psychosocial and spiritual support. Subscales that did not improve were physical functioning, because they were mostly frail and old patients; diarrhea; and financial difficulties, probably because BTcP can impair work performance [32]. These 3 items improved but without statistically significant differences from the baseline. Moreover, more than 80% of both patients and physicians reported some grade of global impression of improvement. However, physicians had a more optimistic impression about the patient’s improvement than the patients themselves. Only 3% of physicians but more than 26% of patients considered that the improvement was minimal, whereas 40% of physicians but only 12% of patients answered “very much improved.” This finding could reflect the different expectations on analgesic treatment between physicians and patients.

Regarding analgesic efficacy and tolerability of transmucosal fentanyl in patients with BTcP, previous studies did not find any differences by age groups except a slower onset of analgesia in patients ≥ 65 years old [33, 34]. However, in spite of the analgesic effect of fentanyl, no conclusive change in QoL of patients ≥ 65 years was shown [34]. In this study, QoL improvement after individualized BTcP therapy was less likely in patients > 65 years and in hospitalized patients. The study data do not allow us to know specifically why these patients are less likely to improve their QoL. A probable reason is that hospitalized patients would have a worse general condition, especially if they are older, which would limit the effectiveness of supportive treatment. Some studies on the complexity of palliative care need confirmation that the combination of a high symptom burden, refractory pain, and functional deterioration determines high complexity, which is associated with the uncertainty in the result, the intensification of care, and hospital death [35].

The main limitation of this study is that it was not a clinical trial and it was not aimed to assess efficacy of analgesic drugs in BTcP. However, it showed QoL improvement after individualized BTcP therapy in clinical practice of palliative care units. Moreover, frail and elderly patients were included, which are usually underrepresented in clinical trials.

Individualized BTcP management includes careful design of drug therapy (initial dose, titration, and follow-up), advice on non-pharmacological interventions, and education on the use of ROO medication. Transmucosal fentanyl was the drug most used for BTcP treatment. Sublingual low dosage (67 μg, 100 μg, and 133 μg) and cautious titration were a suitable option in frail and older patients, according to physician judgment. Therefore, these results could help to individualize BTcP management in patients with advanced cancer.

From these results, it can be concluded that individualized BTcP therapy, in the context of a palliative comprehensive support, improves QoL of patients with advanced cancer in clinical practice. However, this improvement is less likely in patients older than 65 years or hospitalized.

## Supplementary Information

ESM 1 (DOCX 19 kb)

## Data Availability

More data are available on request.
