# Supplementary material for: Impact of individualized management of breakthrough cancer pain on quality of life in advanced cancer patients: CAVIDIOPAL study
Source: Support Care Cancer. 2021 Feb 3;29(8):4799–807. doi: 10.1007/s00520-021-06006-1 (PMC8236456; doi:10.1007/s00520-021-06006-1)
Supplement: Supplementary file 1 — (DOCX 19 kb) [file 520_2021_6006_MOESM1_ESM.docx]

**Title:** Impact of individualized management of breakthrough cancer pain on quality of life in advanced cancer patients: CAVIDIOPAL study

**Journal:** *Supportive Care in Cancer*

**Authors**

Albert Tuca Rodríguez, ORCID iD 0000-0002-4633-5856, Hospital Clínic i Provincial, Barcelona, Spain.

Miguel Núñez Viejo, ORCID iD 0000-0001-6361-2133, Complejo Hospitalario Universitario de Orense, Spain.

Pablo Maradey, Hospital de Sant Joan de Déu, Palma de Mallorca, Spain.

Jaume Canal-Sotelo, ORCID iD 0000-0002-9150-5228, Hospital Universitari Arnau de Vilanova, Lleida, Spain.

Plácido Guardia Mancilla, ORCID iD 0000-0002-2629-7522, Hospital San Cecilio, Granada, Spain.

Sonia Gutiérrez Rivero, Hospital Universitario Virgen del Rocío, Sevilla, Spain.

Inmaculada Raja Casillas, Hospital Virgen de la Salud, Toledo, Spain.

María Herrera Abián, Hospital Universitario Fundación Jiménez Díaz, Madrid, Spain.

Cristina López Bermudo, ORCID ID 0000-0002-9354-6859, Medical Department, Angelini Farmacéutica, SA, Barcelona, Spain.

*Corresponding author*

Albert Tuca Rodríguez

atuca@clinic.cat

| **TABLE S1. Individualized opioid therapy and evolution of background pain and BTcP characteristics in patients with advanced cancer during the study (V0 –V90)** | | | | | | | |
| --- | --- | --- | --- | --- | --- | --- | --- |
|  | V0 | | V8 | V15 | | V28 | V90 |
|  |  | | | Mean (SD)** | | |  |
| Intensity of background pain (VAS)* | 4.0 (1.9) | 2.9 (2.1) | | 2.4 (1.8) | 2.0 (1.6) | | 1.8 (1.7) |
| Intensity of BTcP (VAS)* | 8.0 (1.0) | 5.8 (2.1) | | 4.9 (2.3) | 4.6 (2.4) | | 4.0 (2.4) |
| Number of BTcP episodes/previous 24h | 3.6 (1.6) | 2.6 (1.4) | | 2.2 (2.0) | 1.5 (1.2) | | 1.5 (1.1) |
| Duration of BTcP episodes (minutes) | - | 20.7 (13.0) | | 19.5 (16.6) | 17.4 (14.6) | | 13.6 (11.4) |
| Time until BTcP relief after treatment (minutes) | - | 10.2 (5.3) | | 9.2 (6.1) | 8.3 (5.6) | | 7.5 (5.2) |
| Time with uncontrolled BTcP/previous 24 h (minutes) | 99.0 | 53.8 | | 42.9 | 26.1 | | 20.4 |
|  |  |  | | N (%) | | |  |
| Change in background pain therapy | - | 45 (45.9) | | 23 (23.2) | | 18 (18.2) | n.a. |
| Change of opioid dose | - | 34 (34.3) | | 20 (20.2) | | 13 (13.1) | n.a. |
| Change of opioid drug | - | 11 (11.1) | | 3 (3.0) | | 5 (5.1) | n.a. |
| Change in BTcP therapy | - | 38 (38.8) | | 15 (15.8) | | 12 (12.6) | 2 (2.1) |
| Change of ROO dose | - | 26 (26.3) | | 13 (13.1) | | 11 (11.1) | 2 (2.0) |
| Change of type of ROO | - | 12 (12.1) | | 2 (2.0) | | 1 (1.0) | 0 |
| By adverse event | - | 2 (2.0) | | 1 (1.0) | | 0 | 0 |
| By insufficient analgesia | - | 31 (31.3) | | 14 (14.1) | | 11 (11.1) | 2 (2.0) |

BTcP, breakthrough cancer pain; n.a., data not available; ROO: rapid onset opioid; VAS, visual analog scale.

*p < 0.001 from V0 to V90.

**Values of N could be different between visits and variables of background pain and BTcP.
